# Effect of Chitosan and Liposome Nanoparticles as Adjuvant Codelivery on the Immunoglobulin G Subclass Distribution in a Mouse Model

**DOI:** 10.1155/2017/9125048

**Published:** 2017-07-05

**Authors:** Agus Haryono, Korrie Salsabila, Witta Kartika Restu, Sri Budi Harmami, Dodi Safari

**Affiliations:** ^1^Research Center for Chemistry Indonesian Institute of Sciences, Kawasan Puspiptek Serpong, Tangerang, Indonesia; ^2^Eijkman Institute for Molecular Biology, Jakarta, Indonesia

## Abstract

**Background:**

We investigate the immunogenic properties of chitosan and liposome nanoparticles as adjuvant codelivery against a commercial pneumococcal conjugate vaccine (PCV) in an animal model.

**Methods:**

The chitosan and liposome nanoparticles were prepared by ionic gelation and dry methods, respectively. The PCV immunization was performed intradermally in the presence of adjuvants and booster injections which were given without an adjuvant. The Quil-A® was used as a control adjuvant. The ELISA was performed to measure the antibodies against pneumococcal type 14 polysaccharide (Pn14PS).

**Results:**

The level of total antibodies against Pn14PS antigen was no different between the mouse groups with or without adjuvant codelivery. Codelivery of the PCV with chitosan nanoparticles as well as the Quil-A adjuvant elicited IgG1, IgG2a, IgG2b, and IgG3 antibodies. Meanwhile, codelivery of liposome nanoparticles elicited mainly IgG1 antibodies against the Pn14PS.

**Conclusions:**

The chitosan and liposome nanoparticles as adjuvant codelivery were successfully synthesized. These nanoparticles have different shapes in particle formation, liposome nanoparticle with their unilamellar shape and chitosan nanoparticles in large shape due to the aggregation of small-size particles. Codelivery of chitosan nanoparticles has more effect on the IgG subclass antibody production than that of liposome nanoparticles in a mouse model.

## 1. Introduction

Nanoparticles are increasingly used as adjuvant vaccine formulations due to their biocompatibility, ease of manufacture, and the opportunity to tailor their size, shape, and physicochemical properties [1]. Chitosan is a nontoxic, biocompatible, biodegradable, natural polysaccharide whose the molecular weight is between 3800 and 20,000 Daltons and the degree of deacetylation ranges from 60% to 100% [2]. Due to its useful properties, chitosan offers advantages for adjuvant and vaccine delivery systems [3, 4]. Sáenz et al. reported that the chitosan formulation improves the immunogenicity of a peptide-based vaccine and was able to induce an immune response mediated by IgG2a [5]. Liposomes, sphere-shaped vesicles with particle sizes ranging from 30 nm to several micrometers consisting of one or more phospholipid bilayers, were characterized to deliver active molecules to the site actions, and at present, several formulations are in clinical use [6, 7]. Liposomes are structurally suitable to make nanoparticles biocompatible and offer a clinically proven, versatile platform for the further enhancement of pharmacological efficacy [8]. It was reported that a cationic liposome-forming lipid worked as potential adjuvants for protein subunit vaccines. This potential adjuvant also appeared to favour a stronger Th1 immune response [9].

In this present study, we investigate the immunogenic properties of chitosan and liposome nanoparticles as adjuvant codelivery against a commercial pneumococcal conjugate vaccine (PCV) in an animal model.

## 2. Materials and Methods

### 2.1. Adjuvants and Vaccine

Low molecular weight chitosan (C_8_H_15_NO_6_)_n_ powder with 75% degree of deacetylation and soybean phosphatidylcholine were purchased from Sigma-Aldrich, USA. The Quil-A saponin adjuvant was a gift from Dr. Erik B. Lindblad and Brenntag Biosector, Denmark. The 13-valent pneumococcal conjugate vaccine (PCV13; Prevnar®; Pfizer Inc.) as a PCV antigen model was commercially acquired. The PCV13 contains the capsular polysaccharide antigens of *Streptococcus pneumoniae* serotypes 1, 3, 4, 5, 6A, 6B, 7F, 9V, 14, 18C, 19A, 19F, and 23F which individually conjugated to the nontoxic diphtheria toxin mutant CRM_197_.

### 2.2. Preparation of Adjuvant Nanoparticles

Chitosan nanoparticles were prepared by dissolving 0.2 g powdered chitosan in 0.5% acetic acid and mixing for 24 hours. After that, six drops of 10 M sodium hydroxide were added until the pH reached to 4.6–4.8. The obtained mixture was added with 0.5% sodium tripolyphosphate with a mole ratio of 3 : 1 for chitosan and sodium tripolyphosphate. The last step was homogenization of the mixture for 15 minutes with a stirring speed of 500 rpm [10–12].

35 mg liposome nanoparticles were prepared by dissolving phosphatidylcholine into 3 mL chloroform and then by evaporation. Phosphate buffer saline (pH 7.4) was added to modulate the thin layer of a lipid film [13]. Later on, this mixture was vortexed and sonicated for 15 minutes. The obtained liposomes were stored in a refrigerator until use.

Characterization of nanoparticles was carried out by a nanoparticle analyzer (Beckman Coulter Delsa) and transmission electron microscope (TEM) (JEM-1400, JEOL, Japan) for the mean particle size and the morphology of nanoparticles, respectively.

### 2.3. Mouse Immunization

The immunization study was approved by the Animal Care and Use Committee of PT. Bimana Indomedical, Bogor, Indonesia. Inbred 6-week-old female BALB/c mice were maintained at the Animal Laboratory of PT. Bimana Indomedical, Bogor, Indonesia. Five mice per group were immunized intradermally with the PCV diluted in saline to 1 : 10 (100 *μ*L per mouse) [14] in mixture with adjuvants (0.1–0.2 mg/mL): chitosan nanoparticles, liposome nanoparticles, and Quil-A adjuvant [15]. The Quil-A adjuvant which contains the water-extractable fraction of saponins from the South-American tree, *Quillaja saponaria* Molina, has been used to improve the immunogenicity of a synthetic carbohydrate-based vaccine [15]. Saline (0.9% [wt/vol] NaCl in water) was used as a negative control. A booster of the PCV antigen diluted in saline to 1 : 10 (100 *μ*L per mouse) was given on day 35 without an adjuvant. Blood samples were taken one week after the booster immunization.

### 2.4. Measurement of Antibodies by ELISA

The enzyme-linked immunosorbent assay (ELISA) was performed to measure the antibodies to pneumococcal type 14 polysaccharide (Pn14PS) antigen, one of the polysaccharide antigens included in the PCV, as described previously [16]. Briefly, serially diluted sera were incubated for 1 h at 37°C in flat-bottom plates (Corning Inc., Corning, NY, USA), coated with 100 *μ*L of purified polysaccharide types 14 and 23 (5 *μ*g/mL). After coating, the plates were blocked with 2% gelatin (Sigma) and then washed and horseradish peroxidase-conjugated goat anti-mouse IgG (1 : 5000) (Silenus, Amrad Operations PTY LTD, Australia) or IgG subclass-specific antibodies (1 : 5000) (1 mg/mL; Abcam) were added and incubated for 1 h at 37°C. A ready-to-use TMB (3,3′,5,5′-tetramethylbenzidine) with HRP (horseradish peroxidase) substrate (Life Technologies) was added to visualize the amount of bound peroxidase. The reaction was stopped by the addition of 0.5 M H_2_SO_4_. Optical density (OD) values were obtained with a microtiter plate spectrophotometer (Tecan Infinite F200, Switzerland) at 450 nm. Antibody titers were expressed as the log10 of the dilution giving twice the OD obtained for control mice.

## 3. Results

### 3.1. Characterization of Nanoparticles

**Figure 1:**
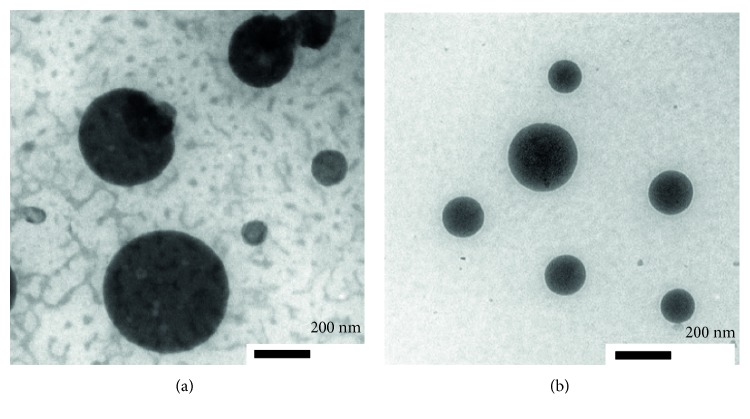
Morphology of nanoparticles (scale bar: 200 nm): TEM image of chitosan nanoparticles (a) and TEM image of liposome nanoparticles (b).

In this study, chitosan nanoparticles were prepared by the ionic gelation method with the interaction with the small anionic molecule tripolyphosphate (TPP). The TPP was used to prepare chitosan nanoparticles due to its characteristics being nontoxic, multivalent, and able to form gels through ionic interactions [17, 18]. The formation of nanoparticles was indicated by the appearance of an opalescence, and it was confirmed by dynamic light scattering which indicated a size of 46.1 nm [19]. Liposome nanoparticles that were synthesized by the method of Kirby and Gregoriads resulted in nanoparticle size of 31.4 nm. This result showed that the obtained liposomes reach the required particle size. Morphology of liposome and chitosan nanoparticles was investigated by using a TEM. The morphology of liposome nanoparticles appeared as unilamellar spherical shape (Figure 1(a)). Meanwhile, the morphology of chitosan nanoparticles is confirmed that the particles are small and individual in which they had a tendency to fuse. This fusion resulted in larger size particles due to the aggregation of these small particles (Figure 1(b)).

### 3.2. Effect of Adjuvants on Antibody Response and Anti-Pn14PS IgG Subclass Distribution

**Figure 2:**
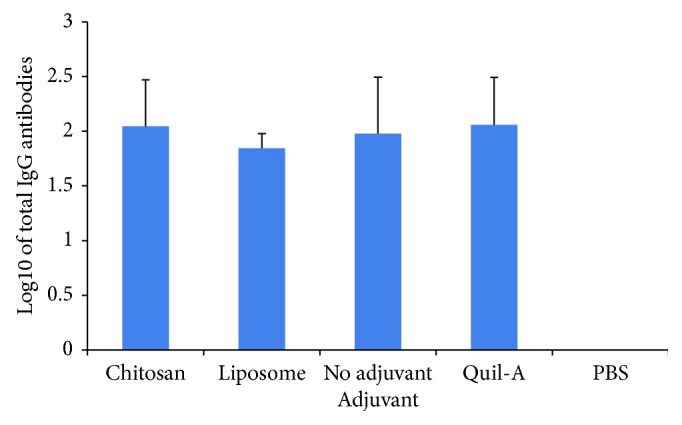
Total IgG antibody titers recognizing a series of pneumococcal polysaccharides of serotype 14. The group of mice (*n* = 5) was immunized with pneumococcal conjugate with a series of adjuvant coadministration at the primary injection. Sera were collected one week after the booster injection which was given without an adjuvant. Saline immunization served as the negative control. Antibody titers were expressed as the log10 of the dilution giving twice the absorbance value corrected by buffer.

Mouse groups were immunized with PCV antigen with a series of adjuvants at day 0, and booster injection was done with the same dose of the PCV without adjuvant codelivery at day 35. The level of antibodies against pneumococcal type 14 polysaccharide antigens after the booster injection was determined by ELISA. The level of antibody against pneumococcal type 14 polysaccharide has the same pattern between the groups of mice with adjuvants and without adjuvant codelivery (Figure 2).

**Figure 3:**
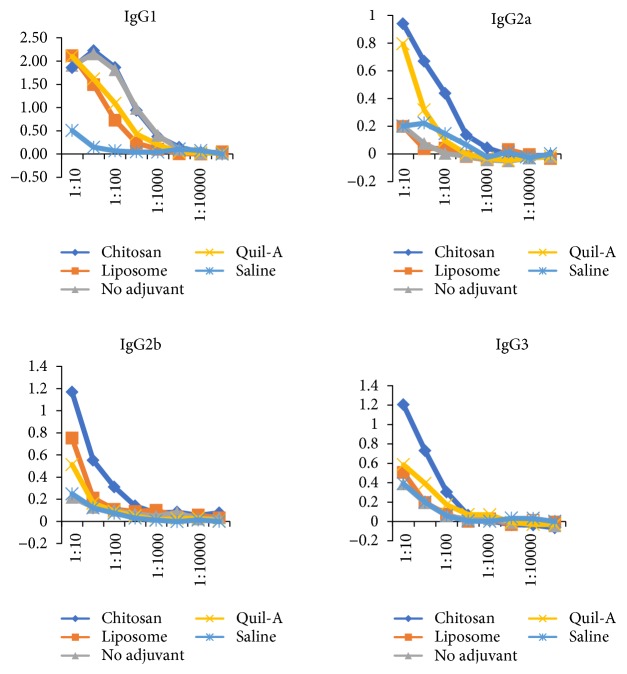
Anti-pneumococcal type 14 polysaccharide (anti-Pn14PS) IgG antibody subclass distribution. Mouse sera (ranging from 1:10 to 1:10000) were collected after the booster immunization. ELISA was performed to measure the anti-Pn14PS IgG antibody subclass distribution: IgG1, IgG2a, IgG2b, and IgG3. The level of antibodies is expressed as optical density (OD) at 450 nm.

The diversification of anti-pneumococcal type 14 antibodies to other IgG subclasses was investigated after the booster injection. The PCV immunizations without an adjuvant mainly elicit IgG1 antibodies. Meanwhile, the mouse group with an adjuvant elicits the IgG subclass antibodies (Figure 3()). The mouse group with the chitosan adjuvant evoked anti-Pn14PS IgG antibodies IgG1, IgG2a, IgG2b, and IgG3 so do the group of mice with the Quil-A adjuvant as the positive control (Figure 3()), while the liposome adjuvant could not show the diversification of IgG isotypes and mainly induces anti-Pn14PS IgG1.

## 4. Discussion

In this study, we observed that liposomes presented morphologically unilamellar spherical shape. Meanwhile, chitosan itself showed as the individual particle that has a tendency to fuse with each other creating larger size particles due to the aggregation inside it. The aggregation might be influenced by the conformation of the random coil in chitosan solution [20]. The obtained nanosize range as particles in those materials could make those to be used as a delivery system, here as an adjuvant. Many other TEM studies also indicated that the particle size, vesicle shape, and lamellarity of liposomes may be different due to the process of preparation [21].

In this study, the effect of adjuvant codelivery was shown by the diversification of anti-Pn14PS IgG subclasses. Previously, it was reported that the Quil-A adjuvant induced IgG diversification and elicited a broader anti-Pn14PS IgG subclass antibody spectrum [15]. We found that antibodies against type 14 polysaccharides between mouse groups with and without adjuvant codelivery were not significantly different. In this study, we also observed that PCV immunization with codelivery of chitosan nanoparticles significantly enhanced anti-Pn14PS IgG subclass responses. The IgG subclasses (IgG1, IgG2a, IgG2b, and IgG3) in mice immunized with the chitosan adjuvant show the same pattern as those in the Quil-A group as the positive control. Previously, Wen et al. [22] observed that mouse immunization with ovalbumin (OVA) and chitosan nanoparticles elicited a balanced Th1/Th2 immune response as indicated by the significant increases in IgG1, IgG2a, and IgG2b antibody subclasses.

We also found that liposome adjuvant immunization showed a low level of anti-Pn14PS IgG antibodies compared with other groups. The liposome adjuvant leads to an immune response dominated by IgG1. Previously, Korsholm et al. reported that one of the major drawbacks of liposomes as vaccine delivery vehicles has been their instability [23]. Liposomes are versatile delivery systems for antigens, and they can carefully be customized towards desired immune profiles by combining them with immunostimulators and optimizing their composition, physicochemical properties, and antigen-loading mode [24].

Our data suggest that chitosan and liposome nanoparticles induced a Th2 (IgG2a, IgG2b, and IgG3) response rather than a Th1 (IgG1) response. In mice, immunoglobulin G1 (IgG1) is associated with a Th2 response, while a Th1 response is associated with the induction of IgG2a, IgG2b, and IgG3 antibodies [25].

## 5. Conclusion

The chitosan and liposome nanoparticles as adjuvant codelivery in antibody production of mice were successfully synthesized. These nanoparticles have different shapes in particle formation, liposome nanoparticle with their unilamellar shape and chitosan nanoparticles in large shape due to the aggregation of small-size particles. Even though the nanometer size was achieved by both nanoparticles, the measurement of antibody production was different from both of them. In producing subclass antibody in a mouse model towards a commercial PCV, the codelivery of chitosan nanoparticles showed more effect compared to that of liposome nanoparticles. The obtained result due to the use of chitosan as adjuvant codelivery broadens the advantageous properties of chitosan as a biomaterial used in medicinal fields.
